# Three-Dimensional Modelling inside a Differential Pressure Laminar Flow Bioreactor Filled with Porous Media

**DOI:** 10.1155/2015/320280

**Published:** 2015-08-02

**Authors:** Birgit Weyand, Meir Israelowitz, James Kramer, Christian Bodmer, Mariel Noehre, Sarah Strauss, Elmar Schmälzlin, Christoph Gille, Herbert P. von Schroeder, Kerstin Reimers, Peter M. Vogt

**Affiliations:** ^1^Department of Plastic, Hand and Reconstructive Surgery, Hannover Medical School, Carl-Neuberg-Straße 1, 30625 Hannover, Germany; ^2^Biomimetics Technologies Inc., 191 Ellis Avenue, Toronto, ON, Canada M6S 2X4; ^3^8 Selden Street, Newton, MA 02468, USA; ^4^Habsburger Allee 56 B, 76767 Hagenbach, Germany; ^5^Colibri Photonics GmbH, Am Mühlenberg 11, 14476 Potsdam, Germany; ^6^Toronto Western Hospital, University of Toronto, East Wing 2nd Floor, 399 Bathurst Street, Toronto, ON, Canada M5T 2S8

## Abstract

A three-dimensional computational fluid dynamics- (CFD-) model based on a differential pressure laminar flow bioreactor prototype was developed to further examine performance under changing culture conditions. Cell growth inside scaffolds was simulated by decreasing intrinsic permeability values and led to pressure build-up in the upper culture chamber. Pressure release by an integrated bypass system allowed continuation of culture. The specific shape of the bioreactor culture vessel supported a homogenous flow profile and mass flux at the scaffold level at various scaffold permeabilities. Experimental data showed an increase in oxygen concentration measured inside a collagen scaffold seeded with human mesenchymal stem cells when cultured in the perfusion bioreactor after 24 h compared to static culture in a Petri dish (dynamic: 11% O_2_ versus static: 3% O_2_). Computational fluid simulation can support design of bioreactor systems for tissue engineering application.

## 1. Introduction

Bioreactor designs for bone tissue engineering applications often use perfusion models where fluid shear stresses through porous scaffolds activate biological processes such as cell proliferation and differentiation [1–5]. The shear stresses acting on the cultivated cells depend not just on the geometry and internal structural shape of the scaffolds, but also on the whole reactor vessel design, the position of the scaffold within, and the way and mode of perfusion [5–7]. Most applications use cylindrical-shaped scaffolds made from various materials such as ceramic, polyester, collagen, or silk matrices, which can be perfused either through their circular or through their lateral surface area [7–13]. Uniform perfusion is desirable for sufficient nutrient and oxygen transport to cells seeded inside the scaffolds. Increase in cell number during tissue growth leads to changes in scaffold permeability and also changes in flow and pressure distribution [5, 14]. In addition to cell growth, morphological changes, cell migration, and continuous perfusion can lead to changes in matrix fiber arrangement and remodelling that also influence the physical properties of the internal scaffold [15].

Besides external forces such as shear stresses, pressure, and gravity, the concentration of oxygen is an important factor of the physiologic environment during bone growth and repair. Dissolved oxygen concentration has the unit mmol/L or mg/L, but, in the physiologic context, the unit is often expressed as % where 21% means that a solution is air-saturated, which corresponds to 6,7 mg/L oxygen at 37°C and 1013 hPa [16]. Low oxygen concentrations are found in the stem cell reservoir in the bone marrow (2–7%), in avascular tissues such as cartilage (1–6%), or at sites of infections or acute injury such as fractures (1%) [17, 18]. Higher oxygen concentrations (5–10%) are required during the mineralization process in bone formation and repair, when small vessels start to sprout into the cartilaginous ground substance or nonmineralized callus. The direct interaction between vascular endothelial cells and bone-forming osteoblasts leads to deposition of osteoid and begin of the mineralization process [19]. In cell culture experiments* in vitro*, a reduced oxygen tension has been accompanied by an increased proliferation rate and a decreased differentiation capacity of stem and osteoprogenitor cells compared to normoxic culture conditions [20–26]. Therefore, it is of great importance to monitor oxygen concentration inside bioreactors and even more inside scaffolds during the cultivation process.

We have recently designed a differential pressure laminar flow bioreactor based on a two-dimensional CFD (computational fluid dynamics) model for flow and pressure control to support cell survival and tissue growth by prevention of high shear forces and pressure peaks [27–29]. Biological testing revealed beneficial conditions for stem cell growth and differentiation into bone tissue when cultured inside the bioreactor system [15, 30]. A further system upgrade was achieved by implementation of an oxygen sensor system for real-time, noninvasive monitoring of oxygen concentration within a tissue-engineered scaffold during culture in the bioreactor [31]. The aim of the present study was to focus our simulation on the reactor vessel itself and apply a three-dimensional CFD model to our system and evaluate flow and pressure distribution and oxygen content during experimental conditions.

## 2. Materials and Methods

Star-CCM+ program (version 8.04) from CD-Adapco was used for three-dimensional flow simulation. CAD data set from the bioreactor model was imported and converted, and a polyhedral prism mesh with 1.2° million cells and 6.9° million faces was created by the program-innate surface wrapper. To describe the flow inside the bioreactor, Reynolds-averaged Navier-Stokes equations were applied for liquid media. The inlet of the bioreactor was set as velocity with 8.77*∗*10^−4^ m/s which corresponds to a pump rate of 1 mL/min used experimentally, the outlet as pressure, and the flow as steady, segregated flow with water density. The culture medium was treated as incompressible Newtonian fluid with the viscosity of water at 37°C. Scaffold properties used for simulation are stated below. Convergence criterion was set at 0.001 for continuity, *x*-velocity, *y*-velocity, and *z*-velocity. The number of iterations was set at 500.

### 2.1. Scaffold Properties

The scaffold was simulated with a constant porous medium model using hybrid LSQ (least squares) Green-Gauss model. Parameters for modelling flow through porous media are summarized in Table 1 which were derived from previous work [29] and were converted to units used by the Star-CCM+ program.

For unit conversion, the porous viscous resistance [m^−2^] was multiplied by the viscosity of the fluid (water at 37°C, which is 6.915 *∗* 10^−4^ Pa*∗*s = 6.915 *∗* 10^−4^ kg*∗*m^−1^ *∗*s^−2^) to get the unit [kg*∗*m^−3^ *∗*s^−1^]. The porous inertial resistance [m^−1^] was divided by 2 and multiplied with the fluid density (for water, which is 992.2 kg*∗*m^−3^) to convert to the unit [kg*∗*m^−4^].

The different permeabilities represent a collagen matrix (reported permeability in literature *κ* = 10^−9^ up to 10^−13^) with decreasing permeability during cell growth [32, 33].

For experimental testing in the bioreactor, we used MatriDerm (MedSkin Solution Suwelack, Billerbeck, Germany), which is a collagen-elastin matrix with a dense inner fiber structure and porous sizes between 20 and 50 and up to 100 and 150°*μ*m.

### 2.2. Model

The momentum equations considered for the porous medium are described by Batchelor [34]:

(1) $$\begin{array}{c} \frac{\partial }{\partial t}\left(\;\rho \vec{\nu }\;\right)+\nabla \cdot \left(\;\rho \vec{\nu }\vec{\nu }\;\right)=-\nabla p+\nabla \cdot \left(\;\bar{\bar{\tau }}\;\right)+\rho \vec{g}+\vec{F}, \end{array}$$

where *p* is the static pressure, $\bar{\bar{\tau }}$ is the stress tensor, $\rho \vec{g}$ is the product of density and gravitational constant vector (*ρ* is the density and *g* is the gravitational constant), and $\vec{F}$ are the gravitational body force and external body forces (e.g., that arise from interaction with the dispersed phase).

The stress tensor $\bar{\bar{\tau }}$ is given by

(2) $$\begin{array}{c} \bar{\bar{\tau }}=\mu \left[\;\left(\;\nabla \vec{\nu }+\nabla {\vec{\nu }}^{T}\;\right)-\frac{2}{3}\nabla \cdot \vec{v}I\;\right], \end{array}$$

where *μ* is the molecular viscosity, *I* is the unit tensor, and the second term on the right hand side is the effect of volume dilation.

For 3D geometries, conservation equations are given by

(3) ∂∂tρvx+1r∂∂xrρvxvx+1r∂∂rrρvrvx=−∂p∂x+1r∂∂xrμ2∂vx∂x−23∇·v→+1r∂∂rrμ∂vx∂r+∂vr∂x+Fx,∂∂tρνr+1r∂∂xrρνxνr+1r∂∂rrρνrνr−∂p∂r+1r∂∂xrμ∂νr∂x+∂νx∂r+1r∂∂rrν2∂νr∂r−23∇·ν→=2μνrr2+23μr∇·ν→+ρνz2r+Fr,

where

(4) $$\begin{array}{c} \nabla \cdot \vec{\nu }=\frac{\partial \nu_{x}}{\partial x}+\frac{\partial \nu_{r}}{\partial r}+\frac{\nu_{r}}{r}. \end{array}$$

The porous media term is composed of two parts: Darcy's, which contains the first part of the right-side equation, and a viscous loss term, which is defined by the second part of the right-side equation:

(5) $$\begin{array}{c} S_{i}=-\left(\;\overset{3}{\underset{j=1}{\sum }}D_{ij}\mu \nu_{j}+\overset{3}{\underset{j=1}{\sum }}C_{ij}\frac{1}{2}\rho \left|\;\nu \;\right|\nu_{j}\;\right), \end{array}$$

where *S*_*i*_ is the source term for the *i*th (*x*, *y*, *z*) momentum equation, |*ν*| is the magnitude of the velocity, and *D* and *C* are prescribed matrices. This momentum sink contributes to the pressure gradient in the porous cell, creating a pressure drop that is proportional to the fluid velocity (or velocity squared) in the cell.

In Star-CCM+, the porous jump conditions are used to model a thin membrane that has known velocity (pressure drop) characteristics. It is essentially a 1D simplification of the porous media model available for cell zones.

The thin porous medium has a finite thickness over which the pressure change is defined as a combination of Darcy's law and an additional inertial loss term:

(6) $$\begin{array}{c} \Delta p=-\left(\;\frac{\mu }{\kappa }\nu +C_{2}\frac{1}{2}\rho \nu^{2}\;\right)\Delta m, \end{array}$$

where *μ* is the laminar fluid viscosity, *κ* is the permeability of the medium, *C*_2_ is the pressure-jump coefficient, *ν* is the velocity normal to the porous face, and Δ*m* is the thickness of the medium.

### 2.3. Mass Flux

The mass flux is the rate of mass flow per unit area [kg*∗*s^−1^ *∗*m^−2^] and is given by

(7) $$\begin{array}{c} \dot{m}=\rho a\cdot v, \end{array}$$

where *ρ* is the density of the fluid, *a* is the area, and *v* velocity of the flow (as a component of magnitude).

### 2.4. Bioreactor Cultivation and Oxygen Monitoring

For biological experiments, human adipose-derived mesenchymal stem cells were derived from fat tissue donors undergoing abdominoplasty after informed consent and in accordance with the guidelines of the Ethical Committee of Hannover Medical School. The full procedure followed standard protocols and has been described elsewhere [35]. Mesenchymal stem cells were cultivated in a cell culture incubator at 37°C and 5% CO_2_ and expanded in standard culture medium (DMEM-F12 (PAA laboratories, US)), supplemented with 5% fetal calf serum, antibiotics, sodium pyruvate, and nonessential amino acids (all Biochrom, USA). Stem cells were continuously tested for multipotency and stem cell surface markers [36].

**Figure 1:**
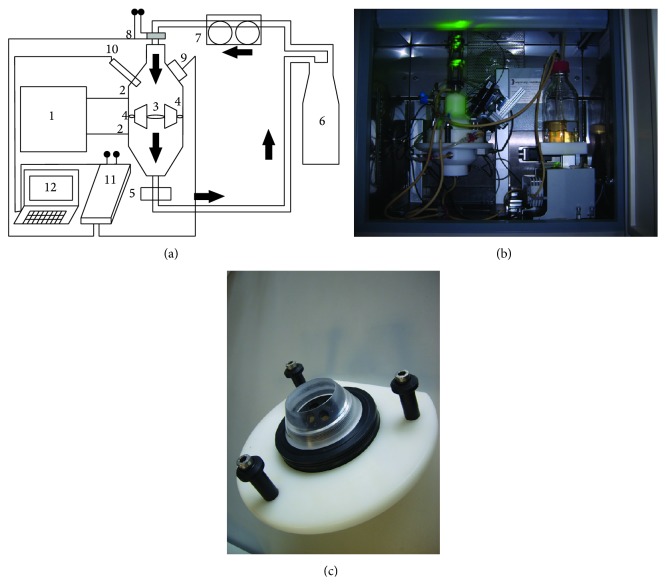
(a) Schematics of differential pressure laminar flow bioreactor system with integrated laser-based oxygen sensor system (from PCT/EP2011/067344/WO 2012/045756). 1: Control box, 2: pressure ports, 3: bioreactor vessel with scaffold holder, 4: integrated bypass system, 5: sampling probe (for medium analysis), 6: culture medium reservoir, 7: peristaltic pump, 8: laser light with filters and lenses, 9: photomultiplier (light detector), 10: temperature sensor, 11: OPAL system with built-in sinusoidal frequency generator and lock-in amplifier, 12: computer with software, and** →**: medium flow direction. (b) Photo of system. (c) Scaffold holder suitable for cultivation of up to seven cylindrical scaffolds.

The bioreactor was equipped with a laser-based oxygen measurement system called “OPAL,” which measures oxygen-dependent phosphorescence lifetime of microbeads (50 *μ*m diameter), and uses a two-frequency modulation technique to eliminate interference by background fluorescence [37]. The system, which actually is intended to be used with fluorescence microscopes, has been described in detail previously [37–39]. In order to detect the signal of the spherical oxygen microsensors, the photomultiplier unit was linked to a camera objective and placed in front of a window, which had been integrated in the bioreactor wall (see Figure 1).

For experiments, cylindrical collagen scaffolds were prepared with phosphorescent microbeads which were placed centrally inside the scaffolds and fixated with 20 *μ*L of fibrin glue. Scaffolds were seeded with 1.3 *∗* 10^7^ adipose mesenchymal stem cells of which 5% were glued together with the microbeads in the center of the scaffold, and the remaining 95% were injected randomly into the cylinder. Wetted collagen scaffolds had a diameter of 10 mm and a height of 15 mm. Cell-seeded scaffolds were then transferred inside glass Petri dishes with a diameter of 8 cm and a height of 3 cm and cultured statically in a standard incubator at 37°C and 5% CO_2_. For dynamic cultivation, scaffolds were placed inside the bioreactor and cultivated at 37°C under continuous perfusion with addition of HEPES buffer solution (PAA laboratories, US) to the culture medium in order to maintain a stable pH. At the end of experiments, scaffolds were fixed in formalin-based solution and processed for microscopy analysis (conventional staining, fluorescence staining, and scanning electron microscopy).

## 3. Results

**Figure 2:**
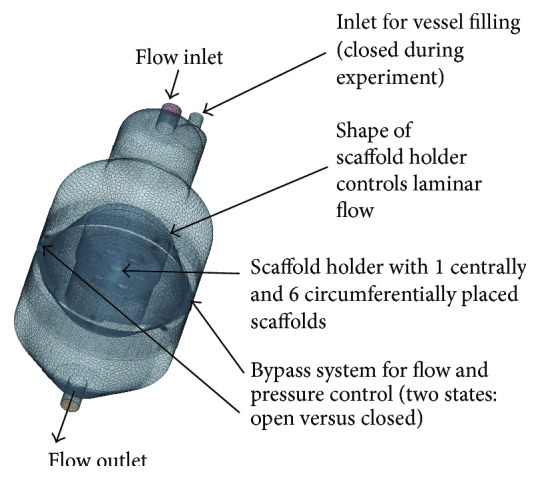
Mesh of the bioreactor created with the Star-CCM+ program.

The laminar flow bioreactor and the oxygen sensor system are being presented in Figure 1.  Figure 1(a) shows a schematic view of the whole setup, Figure 1(b) shows the bioreactor during an experimental course, and Figure 1(c) demonstrates the scaffold holder device suitable for simultaneous cultivation of up to seven cylindrical scaffolds with a diameter of 10 mm. The lateral positioned bypass system, which can be seen in Figure 1(a) number 4, was designed to release pressure build-up and prevent high shear forces inside the scaffolds during cell growth inside the porous matrices. For CFD simulation, a mesh of the bioreactor was created with the surface wrapper and polyhedral volume mesher of the Star-CCM+ program which is presented in Figure 2; here the open bypass systems are marked by an arrow. For the closed bypass model, bypasses were blocked by an interface layer which was integrated in the mesh.

CFD simulations were performed for closed and open bypass systems and scaffolds with different permeability values representing different states of cell growth inside the scaffold as stated in Table 1. There was no convergence at a scaffold permeability of 5 *∗* 10^−13^ and 5 *∗* 10^−12^ when the bypasses in the bioreactor were closed. All other configuration variants showed a solution which converged.

**Figure 3:**
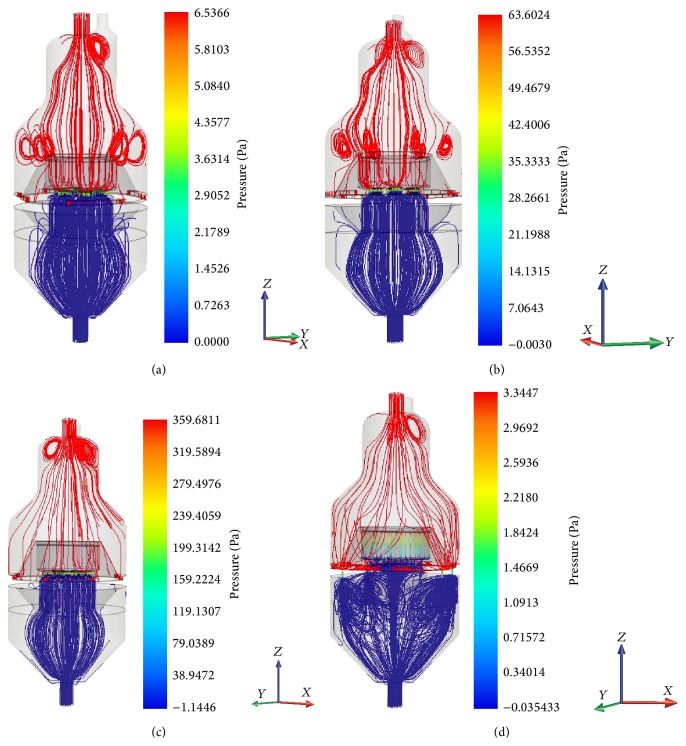
CFD simulation of pressure distribution inside the bioreactor with scaffold permeability of (a) 10^−11^ m^2^, (b) 10^−12^ m^2^, and (c) 10^−13^ m^2^ and closed bypass system demonstrates pressure build-up in the upper part of the culture vessel by decreasing scaffold permeability due to cell growth. Pressure can be released by opening the integrated bypass system (d) (here shown for permeability 10^−13^ m^2^), which results in approximation of pressure values for the upper and lower part of the culture chamber of the bioreactor.

**Figure 4:**
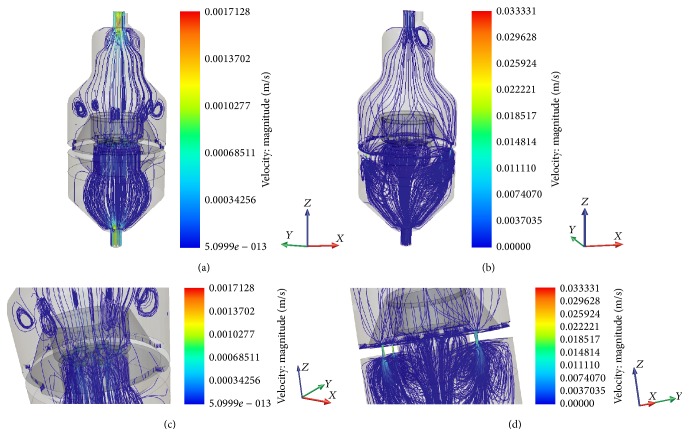
CFD simulation of flow velocity with (a) closed and (b) open bypass system (permeability 10^−12^ m^2^). Closer view on velocity streamlines at the level of the scaffold holder for (c) closed and (d) open bypass configuration (permeability 10^−12^ m^2^).

**Figure 5:**
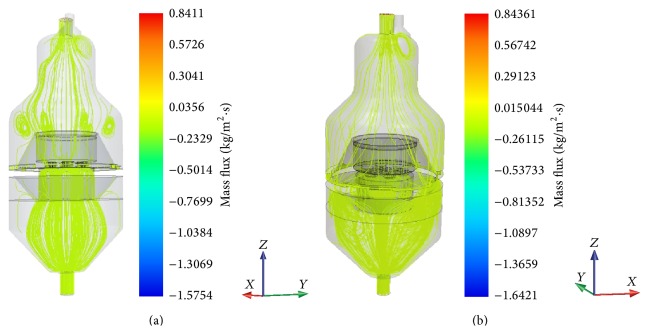
Mass flux for (a) closed and (b) open bypass systems (scaffold permeability 10^−12^).

**Figure 6:**
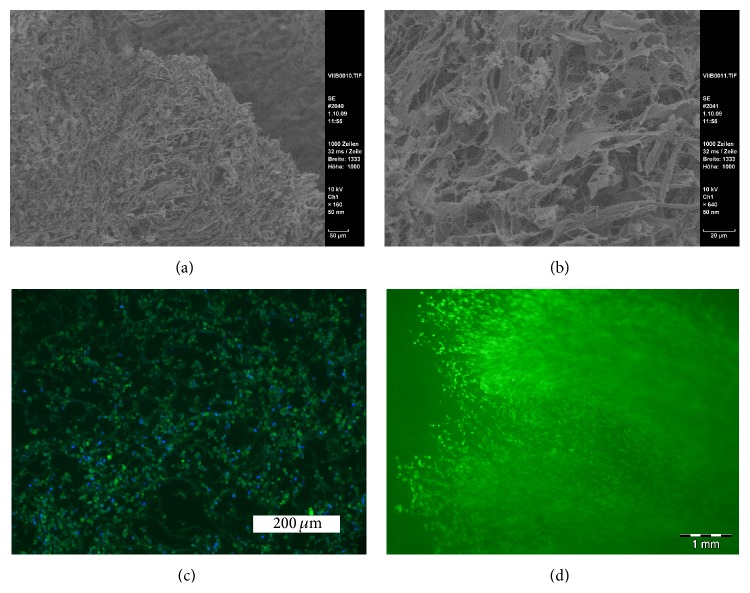
(a and b) Scanning electron microscopy shows the dense fiber network of the collagen matrix with mesenchymal stem cells after 10 days of dynamic culture in the bioreactor. (c) Cross-section of collagen matrix stained with DAPI fluorescence stain shows collagen fibrous network (in green) with adipose mesenchymal stem cells (blue: nuclei of MSC) cultured in the bioreactor after 14 days of culture. (d) Fluorescence microscopy shows vital mesenchymal cells longitudinally aligned after 1-week dynamic culture in perfusion bioreactor (cell vitality stain).

Results of the CFD simulation are presented in Figures 3–6.  Figure 3 shows the pressure distribution in the bioreactor system simulated with various scaffold permeability. With the bypass system of the bioreactor closed, the pressure difference between the upper and lower parts of the culture chamber separated by the scaffold increases with decreasing intrinsic scaffold permeability as seen in Figures 3(a)–3(c). The “pressure drop” accounts for 6.5 Pa for a permeability of 5 *∗* 10^−11^ and 63 Pa for a permeability of 5 *∗* 10^−12^ and increases up to 360 Pa when permeability decreases to 5 *∗* 10^−13^. Opening the bypass system results in pressure release, and pressure values in the upper and lower part of the culture chamber adjust to a pressure difference of about 3.3 Pa, as seen here in Figure 3(d) for the simulation with the lowest scaffold permeability 5 *∗* 10^−13^.

Results of flow velocity analysis are shown in Figure 4 for different configurations. Uniform flow streamlines in the whole bioreactor vessel (Figures 4(a) and 4(b)) and at the level of the scaffold holder (Figures 4(c) and 4(d)) are seen for both the closed bypass and open bypass operations at various scaffold permeability. The flow velocity at scaffold level obtained by CFD modelling was in the range of 10^−10^ down to 10^−14^ m/s for closed or open bypass configuration and represents global values and not velocities in single pores of the scaffold.

Figure 5 shows the results of mass flux (kg m^−2 ^s^−1^) simulation in the bioreactor for closed (a) and open (b) bypass configuration. The results show a homogenous distribution and were in the range between 0.05 and 0.15 kg m^−2 ^s^−1^ for the various permeability values used for the porous media in the model.

Figure 6 demonstrates a scanning electron microscopy picture of the collagen scaffold cultured for 10 days in the bioreactor with adipose-derived mesenchymal stem cells, which can be seen with their cellular extensions inside the dense fibrous network (a + b). Mesenchymal stem cells stayed viable inside the collagen scaffold and aligned in direction of the perfusion stream as seen in fluorescence microscopy in Figures 6(c) and 6(d).

**Figure 7:**
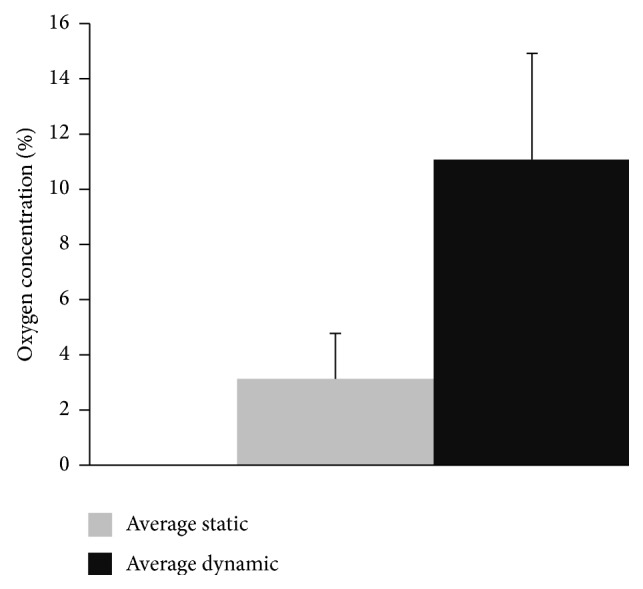
Oxygen concentration measured in statically cultured scaffolds in a Petri dish compared to dynamically cultured collagen scaffolds in a bioreactor after 24 h of seeding with adipose mesenchymal stem cells and further 24 h of static or dynamic culture (statistically significant difference with *p* < 0.05).

Oxygen measurement centrally inside the cell-seeded collagen scaffolds demonstrated a significant difference between statically cultured scaffolds in a Petri dish compared to dynamically cultured scaffolds in the bioreactor as demonstrated in Figure 7. Scaffolds in the bioreactor had a mean central oxygen concentration of 11% after 24 h of perfusion culture, whereas in statically cultured scaffolds an oxygen concentration of 3% was measured (mean results of 8 experiments).

## 4. Discussion

In recent years, computational fluid dynamics has gained more and more interest in the development and optimization of systems for biotechnology such as bioreactors [5, 11, 40, 41]. Our perfusion bioreactor model had been originally developed based on 2D CFD fluid modelling [5] and the resulting prototype [30] was now further analyzed by means of 3D simulation. Star-CCM+ software (version 8.06) from CD-Adapco computed porous media model for single-phase flow and multiphase flow using the superficial velocity porous formulation. The program calculated the superficial phase or mixture velocities based on the volumetric flow rate in the porous region. As stated above, our intention was not to describe the flow through the porous scaffold itself, which would require a different approach, but to examine the flow and pressure characteristics of the bioreactor during operational mode in more detail. Three-dimensional flow characterization of the differential pressure laminar flow bioreactor was feasible for idealized porous media.

Results from our simulation underline the importance of pressure control in the system during cell growth inside the porous scaffold causing decrease in permeability. In our current operating prototype, the pressure control system is set up that a change in differential pressure of 1 mbar = 100 Pa activates the opening of the bypasses [30]. The specific shape of the cultivation vessel and scaffold holder of the bioreactor promotes uniform flow streamlines and prevents turbulences at scaffold levels in a range of settings simulating various states of scaffold architecture and cell and extracellular matrix growth. Observation during bioreactor culture of the collagen scaffold showed changes of the internal structure with alignment of collagen fibers and cells along the flow path-lines during the first ten days of culture and disintegration of the internal scaffold architecture when culture was prolonged for up to four weeks [42]. For simulation of flow in the porous scaffold itself during cell growth, a stable internal architecture would be essential which is, for example, given by ceramic materials or nondissoluble polymers.

Our oxygen measurement data from the interior of the cell-seeded scaffold suggest an improvement of oxygen supply towards the cells during perfusion culture in the bioreactor. Further work is necessary in order to study oxygen distribution inside scaffolds during long-term perfusion cultures and determine its influence on cell growth and differentiation inside tissue-engineered constructs.

## 5. Conclusion

A three-dimensional computational model is presented to characterize flow and pressure distribution inside a perfusion bioreactor prototype. Simulation results underscore the importance of pressure control during changes in porous media permeability caused during cell growth and changes in internal scaffold architecture, which was solved by an internal bypass system for pressure release. Oxygen measurement data suggest an improved oxygen supply for the tissue-engineered constructs in the bioreactor during perfusion culture compared to static cultivation in a Petri dish.

## Figures and Tables

**Table 1:** 

Scaffold	Darcian permeability *κ* [m^2^]	Porous viscous resistance [kg/m^3^ *∗*s]	Porous inertial resistance [kg/m^4^]
A	5*∗*10^−13^	1.23*∗*10^9^	2.95*∗*10^8^
B	5*∗*10^−12^	1.23*∗*10^8^	8.86*∗*10^7^
C	5*∗*10^−11^	1.23*∗*10^7^	2.80*∗*10^7^
D	5*∗*10^−10^	1.23*∗*10^6^	8.86*∗*10^6^
E	5*∗*10^−09^	1.23*∗*10^5^	2.80*∗*10^6^
